# Characterizing the Potential of Phosphonium-Based Ionic Liquids for CO_2_ Capture via Multiscale Modeling

**DOI:** 10.1021/acs.iecr.5c01361

**Published:** 2025-09-02

**Authors:** Sabrina Belén Rodriguez-Reartes, Fèlix Llovell

**Affiliations:** † Department of Chemical Engineering, ETSEQ, Universitat Rovira i Virgili, Avinguda Països Catalans 26, Tarragona 43007, Spain; ‡ Departamento de Ingeniería Química, Universidad Nacional del Sur (UNS), Avda. Alem 1253, Bahía Blanca 8000, Argentina; § Planta Piloto de Ingeniería Química—PLAPIQUI (UNS-CONICET), Camino “La Carrindanga” Km 7, Bahía Blanca 8000, Argentina

## Abstract

Efforts to reduce atmospheric CO_2_ levels focus on minimizing fossil fuel consumption, integrating renewable energy systems, and implementing CO_2_ capture, storage, and utilization. While carbon removal from atmosphere technologies shows promising, industrial efforts prioritize point-source capture for sustainability. A key challenge in solvent design is screening candidates and defining selection criteria. Robust models are needed to characterize their thermophysical behavior for CO_2_ capture. This study adopts a multiscale approach to investigate the CO_2_ gas absorption in phosphonium-based ionic liquids (ILs) with eight anions. The trihexyltetradecylphosphonium cation [P_666,14_]^+^ was paired with various anions due to their known CO_2_ absorption capacity. New molecular models are developed using the soft-SAFT equation, leveraging existing coarse-grain models, analyzing molecule charge distribution through Turbomole-COSMO software for new ILs, and approximating association parameters via density functional theory calculations. Once the models are validated against experimental data, soft-SAFT is used predictively to evaluate the thermophysical properties of these ILs in a wide range of conditions. The analysis encompasses estimations of key process indicators, including the cyclic working capacity, enthalpy of desorption, and CO_2_ diffusion coefficient, ultimately proposing the [P_666,14_]­[Ac] and [P_666,14_]­[bis­(2,4,4-TMPP)] ILs as the most promising solvents. This study validates soft-SAFT as a reliable screening tool for CO_2_ capture solvents and process modeling.

## Graphical abstract

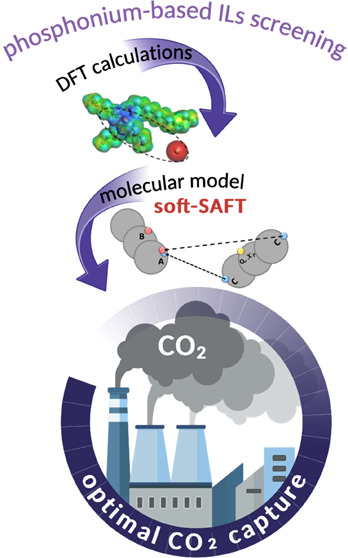

## 1 Introduction

Nowadays, human activities are closely related to environmental issues evidenced in climate change and global warming phenomena, which pose an alert and demand urgent action. Global carbon dioxide (CO_2_) emissions from energy combustion and industrial processes are the main contributors to these issues, reaching 37.4 gigatons (Gt) by 2023.^1^

The deployment of carbon capture, utilization, and storage (CCUS) technologies represents one of the most promising solutions to tackle this problem.^2–4^ CO_2_ can be captured from large point sources, such as power plants or industrial facilities, or directly from the atmosphere. Its subsequent storage and reuse play a critical role in determining the extent of CO_2_ emissions reduction and the potential economic benefits of CCUS facilities.

In industry, the most widely used systems for CO_2_ capture are based on liquid solvents, particularly amines. Their high capture capacity and low cost make them a popular choice in the market.^4^ However, their high volatility and degradation, combined with water vaporization, result in solvent losses. Consequently, their regeneration is energy-intensive and also contributes to equipment corrosion. These challenges sustain the ongoing search for new, efficient solvents in both industry and academia. In this regard, innovative stripper designs enable water recovery via cold rich bypass arrangements, whereas employing water-lean solvents such as ionic liquids effectively minimizes the presence of water in the stripping section.^5,6^

In this context, ionic liquids (ILs) emerge as promising low-volatility solvents, capable of achieving good capture capacities with reduced energy requirements for regeneration while extending the lifetime of absorption circuits. The possibility of synthesizing novel ILs by combining different ions allows tailoring their solvent power.^7–11^ However, a comprehensive characterization is still necessary to identify optimal CO_2_ absorbers, capable to compete with the mature amine technology.^3^

A review of the literature reveals that imidazolium-, pyridinium-, pyrrolidinium-, phosphonium-, and ammonium-based ILs, among others, have been tested as CO_2_ absorbents, demonstrating varying capacities and sorption mechanisms.^7,8,11–13^ Notably, certain phosphonium cation/anion pairings have shown promising results.^8,14–16^

The screening of effective ILs for carbon capture requires the analysis of numerous systems and thermophysical properties. To this end, the use of semipredictive methods, based on quantum, molecular, or group-contribution approaches, to estimate IL-systems properties becomes highly valuable. In fact, a variety of thermodynamic models have been employed in the literature, including COSMO-RS,^8,17,18^ UNIFAC,^19,20^ or SAFT-based equations such as PC-SAFT^21^ and soft-SAFT.^17,22,23^ In particular, soft-SAFT has been successfully applied to evaluate key performance indicators (KPIs) for CO_2_ absorption in ILs and deep eutectic solvents, effectively bridging the molecular and process scales.^22^

This study adopts a multiscale approach to investigate the absorption of CO_2_ gas focused on phosphonium-based ionic liquids with various anions. Eight different ILs are modeled, all formed by the trihexyl­(tetradecyl)­phosphonium cation [P_666,14_]^+^ combined with the following anions: chloride [Cl]^−^, bromide­[Br]^−^, decanoate [Dec]^−^, acetate [Ac]^−^, methylsulfonate [MetS]^−^, bis­(2,4,4-trimethylpentyl)­phosphinate [bis­(2,4,4-TMPP)]^−^, triflate [OTf]^−^, and tetracyanoborate [TCB]^−^. For this purpose, new molecular models for the ILs have been developed within the soft-SAFT^24^ framework, building on existing coarse-grain soft-SAFT models already available for other phosphonium-based ILs.^25^ Molecule charge distributions for new ILs were analyzed using Turbomole-COSMO software, and association parameters were approximated through density functional theory (DFT) calculations. Subsequently, soft-SAFT has been applied to accurately describe and predict the thermodynamic and CO_2_ absorption properties^25^ of these ILs across a wide range of conditions. The molecular models account for specific CO_2_–IL cross-association interactions to accommodate chemisorption phenomena if present. The analysis also includes the estimation of KPIs, including working capacity, solvent required per tonne of CO_2_, heat of regeneration, viscosity, and CO_2_ diffusivity, which were used to systematically compare the solvents and identify the most promising candidates.

This integrative methodology—combining molecular modeling, quantum chemistry, and process-relevant performance indicators—represents a novel and transferable framework to accelerate the screening and selection of ILs for CO_2_ capture applications.

## 2 Methodology

### 2.1 Soft-SAFT Equation of State

Soft-SAFT is a molecular-based equation of State (EoS) developed by Blas and Vega^24^ coming from the original Statistical Association Fluid Theory (SAFT).^25,26^ One of the key aspects of this family of EoSs is the addition of a specific association term based on the first-order thermodynamic perturbation theory of Wertheim (TPT1), to explicitly account for hydrogen bonding.

Soft-SAFT is expressed as a sum of terms that contribute to the total Helmholtz energy of the system, accounting for a series of intermolecular effects

1 $${A=A}^{\mathrm{id}}{+A}^{\mathrm{ref}}{+A}^{\mathrm{chain}}{+A}^{\mathrm{assoc}}{+A}^{\mathrm{polar}}$$

In eq 1, the term *A* ^id^ corresponds to the ideal gas Helmholtz function, while *A* ^ref^, *A* ^chain^, *A* ^assoc^, and *A* ^polar^ represent the reference, chain, association, and polar terms, respectively. The reference term (*A* ^ref^) in soft-SAFT is based on a Lennard–Jones (LJ) intermolecular potential to consider the interactions between the monomers forming the molecule, being calculated through the equation of Johnson et al.^27^ The LJ intermolecular potential is characterized by the diameter of the LJ spheres, σ, and the energy of interaction between the LJ spheres of the chain, ε/*k*_B_.

The *A* ^chain^ term is formally identical in all SAFT formulations and depends on the radial distribution function of the LJ chain. In particular, soft-SAFT uses the expression of Johnson et al. for LJ chains.^27^ The length of this chain is represented by the molecular parameter *m*. Meanwhile, the *A* ^assoc^ term accounts for hydrogen-bonding interactions through some defined association sites, and adds two essential parameters, *ε* ^HB^/*k*_*B*_ and κ^HB^, representing the site–site association energy and site-bonding volume of the association sites, respectively. Finally, *A* ^polar^, accounts for multipolar (dipole and/or quadrupole) interactions between different segments of the molecule. This term was not present in the original formulation but was later added using the treatment of Gubbins and Twu’s theory^28^ and extended by Jog^29^ for chain fluids. Indeed, it is particularly relevant in this work, along with the association term, as it allows accounting for the quadrupolar moment (*Q*) of carbon dioxide. Jog’s theory also assumes that polar moments are localized within specific segments of the molecular chain, defining *x*_p_ as the fraction of segments in the chain that contains the quadrupole. The quadrupole moment was included in the fitting procedure and compared with the literature value to ensure a value of the same order of magnitude is achieved, while *x*_p_ is predetermined based on physical reasoning. For the particular case of CO_2_, a value of 1/3 was assumed in previous publications^22^ based on the geometry of the molecule, and is also retained here. For the remaining molecular parameters, this work employed an approach that combines quantum-chemical calculations to determine the ILs’ association energies and volumes (see Section 2.2 and Section S.2 in the Supporting information), and a classical procedure to fit *m*, σ, and ε/*k*_B_ to single-phase density data.

The chain, association, and polar terms are formulated specifically for mixtures; therefore, only the reference term requires extension for multicomponent systems. Typically, this is achieved by using the van der Waals one-fluid theory, where the unlike size and energy parameters for the LJ fluid are derived from the generalized Lorentz–Berthelot combining rules, as described in eqs 2 and 3.

2 $$\sigma_{\mathrm{ij}}=\eta_{\mathrm{ij}}\frac{\left(\sigma_{\mathrm{ii}}+\sigma_{\mathrm{jj}}\right)}{2}$$

3 $$\varepsilon_{\mathrm{ij}}=\xi_{\mathrm{ij}}\sqrt{\varepsilon_{\mathrm{ii}}\varepsilon_{\mathrm{jj}}}$$

where η_*ij*_ and ξ_*ij*_ are the adjustable size and energy binary interaction parameters, respectively, between species *i* and *j*. These parameters account for asymmetry and nonideal behavior due to the different nature of the compounds in the mixture. If both η_*ij*_ and ξ_*ij*_ are equal to one, the predictions from pure components are satisfactory, the original Lorentz–Berthelot mixing rules are recovered, and the LJ reference term for the mixtures is calculated without any additional fitting.

For mixtures involving hydrogen-bonding interactions, cross-association between different molecules or functional groups within the same molecule is calculated using combining rules, similar to those shown in eqs 2 and 3. When the interaction energies and volumes between a site type α in component *i* and a site type β in component *j* are needed, the combining rules described in eqs 4 and 5 are applied.

4 $$\varepsilon_{{\alpha \beta ,}_{\mathrm{ij}}}^{\mathrm{HB}}=\sqrt{\varepsilon_{{\alpha \beta ,}_{\mathrm{ii}}}^{\mathrm{HB}}\varepsilon_{{\alpha \beta }_{,\mathrm{jj}}}^{\mathrm{HB}}}$$

5 $$k_{{\alpha \beta ,}_{\mathrm{ij}}}^{\mathrm{HB}}={\left(\frac{\sqrt[{3}]{k_{{\alpha \beta }_{,\mathrm{ii}}}^{\mathrm{HB}}}+\sqrt[{3}]{k_{{\alpha \beta ,}_{\mathrm{jj}}}^{\mathrm{HB}}}}{2}\right)}^{3}$$

The reader is referred to the original soft-SAFT contributions for further details about the different terms of the equation.^24,30^

### 2.2 COSMO-RS Approach

Following previous contributions,^17,31^ the conductor-like screening for realistic solvents approach (COSMO-RS),^32^ implemented in BIOVIA, has been used as a complementary tool to build a realistic setup for the molecular models of the phosphonium ILs. The ion geometry has been first optimized in a continuum solvent that resembles an ideal conductor, using DFT calculations in TURBOMOLE software.^33^ During optimization, the conductor influences the ion’s electronic density, and vice versa. COSMO forms a cavity around the ion and calculates the screening charge density (σ) on its surface. Here, −σ corresponds to the molecular charge density, meaning that a positive σ indicates a negative charge density and vice versa. COSMO-RS then processes the screening charge density by dividing it into segments across the cavity’s surface. At this stage, the distribution of segments with a given σ value can be visualized, resulting in what is called the σ-profile. This qualitative σ-profile information is later used as preliminary information to propose realistic coarse-grained models for the ionic liquids modeled in this work with the soft-SAFT EoS.

### 2.3 Free Volume Theory

In this work, the dynamic viscosities of ionic liquids are described using the Free-Volume Theory, developed by Allal and co-workers,^34^ and integrated with the soft-SAFT framework. This theory describes the transition from a low-density to a high-density state through the sum of two terms: the viscosity of the diluted gas, η_o_, based on the modified Chapman–Enskog theory by Chung et al.,^35^ and Δη, a corrective term for calculations performed for dense fluids. In low vapor pressure fluids, such as ILs, the diluted gas term can be omitted, reducing the expression to the calculation of Δη, as described in eq 6

6 $$\Delta \eta =L_{v}\left(0.1P+10^{-4}\alpha \rho^{2}M_{w}\right)\sqrt{\frac{10^{-3}M_{w}}{3\mathrm{RT}}}\exp\left[B{\left(\frac{10^{3}P+\alpha \rho^{2}M_{w}}{\rho RT}\right)}^{3/2}\right]$$

Δη, more specifically, links the viscosity with the fluid’s microstructure and relates it to the free space available between the molecules, based on an exponential relationship proposed by Doolittle.^36^ As can be seen in eq 6, the final mathematical expression depends on thermodynamic variables, such as density (ρ), pressure (*P*), and temperature (*T*), derived from soft-SAFT in this work. Additionally, three adjustable parameters are required: α, which is linked to the energy barrier for molecular diffusion; *B*, a dimensionless parameter characteristic of free-volume overlap; and *L*_v_, the characteristic length parameter of the molecule. These parameters are typically obtained by fitting to experimental viscosity data, which was the strategy followed in the present work.^37^ Furthermore, in eq 6, *M*_w_ and *R* represent the molecular weight of the species and the universal gas constant.

### 2.4 Key Performance Indicators for Solvent Evaluation

In this study, the performance of solvents is evaluated for precombustion CO_2_ capture, typically conducted at low temperatures (298–313 K) and high pressures (1–3 MPa, total pressure). Solvent regeneration is assumed to occur via depressurization, following the principles of pressure swing absorption (PS) processes, via temperature increase, as in temperature swing absorption (TS), and by temperature–pressure swing (TPS). For the purposes of this study, the flue gas was assumed to consist solely of CO_2_. Although this does not reflect the real composition of industrial flue gas, this simplification is sufficient for a comparative screening of the evaluated solvents. Furthermore, while the effects of impurities and selectivity on solvent performance are significant, they are beyond the scope of this work and therefore not assessed.

The techno-economic feasibility of the CO_2_ absorption process is directly influenced by three main factors: the solvent’s CO_2_ absorption capacity, the effectiveness of gas mass transfer, and the energy requirements for solvent regeneration. Following the criteria proposed by Alkhatib et al.,^22^ a set of KPIs is introduced to evaluate the process. These indicators are derived from engineering calculations and thermophysical data predicted by using the soft-SAFT models developed for phosphonium-based ionic liquids and their binary mixtures with CO_2_. A comparative analysis of the KPIs is then performed to evaluate the potential of these solvents and to identify the most suitable options for process operation.

First, the cyclic working capacity of each solvent is quantified in terms of the moles of CO_2_ captured per mole of solvent consumed, as a measure of the solvents’ CO_2_ absorption competence. The amount of CO_2_ effectively captured from the feed gas (i.e., Δα_CO_2__ (*T*, *P*)) is calculated using eq 7

$$\Delta \alpha_{{\mathrm{CO}}_{2}}\left(T,P\right)=\alpha_{{\mathrm{CO}}_{2}}{\left(T_{a\mathrm{bs}},P_{a\mathrm{bs}}\right)}_{\mathrm{absorption}}-\alpha_{{\mathrm{CO}}_{2}}{\left(T_{\mathrm{des}},P_{\mathrm{des}}\right)}_{\mathrm{remaining}}$$ 7

where α_CO_2__ (*T*_abs_, *P*_abs_)_absorption_ is the amount of CO_2_ absorbed by the solvent at absorption conditions, and α_CO_2__ (*T*_des_, *P*_des_)_remaining_ is the amount of CO_2_ remaining in the solvent after the desorption step.

The diffusion coefficient of CO_2_ in the solvent (*D*_CO_2__), under absorption conditions, is calculated to assess the effectiveness of mass transfer. This coefficient can be derived from the solvent′s thermophysical properties using various approximations.^38,39^ In this study, the Wilke–Chang method is employed, as represented in eq 8

8 $$D_{CO_{2}}=7.4\times 10^{-8}\frac{{\left(\varphi M_{w}\right)}^{0.5}T}{\mu V_{m,CO_{2}}^{0.6}}$$

where *M*_w_ is the molar mass of the solvent in g/mol, μ is the viscosity of the solvent in cP, *T* is the temperature in K, *V*_m_ is the molar volume of CO_2_ in cm^3^/g, and φ is the interaction parameter, set to 7.5 as adopted for ILs in previous studies.^22,38,39^

Finally, the energy requirements for solvent regeneration (*Q*) are assessed, as this step typically represents the higher-energy demand in a separation process. Depending on the type of solvent, regeneration can be driven by either a pressure swing or a temperature swing. Since both approaches are feasible for ionic liquids, we have evaluated both. The energy consumption for the recovery of CO_2_ from the solvent is estimated based on the CO_2_ absorption enthalpy and other thermodynamic properties.

For the pressure swing process (solvent regeneration by pressure decrease at constant temperature), the expression in eq 9 accounts for the total energy demand, comprising the energy required to vaporize CO_2_ from the liquid to the vapor phase (*Q*_des_), and the energy associated with the excess enthalpy (*Q*_ex_) between the streams under absorption and desorption conditions

9 $$Q=Q_{\mathrm{des}}+Q_{\mathrm{ex}}$$

*Q*_des_ is the negative of the enthalpy of dissolution (*H*_diss_)­

10 $$Q_{\mathrm{des}}=-H_{\mathrm{diss}}\left(T_{\mathrm{des}},P_{\mathrm{des}}\right)$$

whereas *Q*_ex_ equals the excess enthalpy

11 $$Q_{\mathrm{ex}}=\Delta H_{\mathrm{ex}}$$

Previous studies^40^ have demonstrated that the contribution of excess enthalpy to the total energy requirements is minimal, typically less than 5% at pressures lower than 1 MPa. As a result, the energy consumption for solvent regeneration by a pressure swing depends almost entirely on the enthalpy of dissolution. *H*_diss_ can be approximated using the Clausius–Clapeyron equation, employing thermophysical data predicted by soft-SAFT, as expressed in eq 8

12 $$-H_{\mathrm{diss}}\approx -H_{\mathrm{abs}}=H_{\mathrm{des}}=R{\left(\frac{\partial \;\ln\;P_{i}^{\mathrm{vap}}}{\partial \left(\frac{1}{T}\right)}\right)}_{x_{i}}$$

For the temperature swing process (solvent regeneration by temperature increase at constant pressure), the energy consumption can be estimated as

13 $$Q=Q_{\mathrm{des}}+Q_{\mathrm{sen}}+Q_{\mathrm{vap}}+Q_{\mathrm{ex}}$$

where the desorption energy (*Q*_des_), the sensible energy (*Q*_sen_), and the heat of vaporization (*Q*_vap_) required to vaporize the solvent are estimated with the following expressions

14 $$Q_{\mathrm{des}}=\frac{{-H}_{\mathrm{diss}}}{M_{CO_{2}}}$$

15 $$Q_{\mathrm{sen}}=\frac{C_{p}\Delta T}{C_{\mathrm{ab}}x{M_{\mathrm{CO}}}_{2}}$$

16 $$Q_{\mathrm{vap}}=\frac{{n_{s}\Delta H}_{s}}{{n_{\mathrm{CO}}}_{2}M_{CO_{2}}}$$

where, −*H*_diss_ is the heat of absorption in kJ mol^–1^ predicted from eq 12, *M*_CO_2__ is the molar mass of CO_2_ in g mol^–1^, *C*_p_ is the solvent isobaric heat capacity in kJ kg^–1^ K^–1^, Δ*T* is the difference between the top and bottom temperatures of the desorption column in K, *C*_ab_ is the solvent loading in mol kg^–1^, *x* is the regeneration ratio, Δ*H*_S_ is the vaporization enthalpy of the solvent, and *n*_s_/*n*_CO_2__ is the molar ratio of solvent and CO_2_ at the top of the desorption column.

The regeneration ratio was obtained considering the ratio between the CO_2_ absorbed at the absorption conditions and the CO_2_ desorbed at the desorption conditions as estimated by soft-SAFT EoS. Furthermore, it is assumed that the desorption operates at 0.1 MPa, and a temperature difference of 60 K is considered. Taking into account that the boiling point of the IL is much higher than the desorption temperature, the latent heat was neglected in this study. Furthermore, the excess heat was also ignored for the reasons previously explained. Thus, the regeneration energy for the temperature swing process just depends on the dissolution and sensible heats.^40–42^ *C*_p_ values for the studied PILs are taken from the literature.

## 3 Molecular Models

The choice of an adequate molecular model to represent the different ILs studied in this work plays a key role in the reliable description of the molecule and the thermophysical behavior of the compounds. In this regard, it is important to establish a proper association scheme and specify the molecular parameter values. The main assumption in the construction of the soft-SAFT coarse-grain models is that cation and anion are treated together using a dual site to represent their association, as illustrated in Figure S1 (Section S1 of the Supporting Information). Due to its dual nature, this site can interact with either positively or negatively charged associative sites. This approach is consistent with previously developed soft-SAFT molecular models for molecular liquids.^23,31,43^ Furthermore, if required, single associative sites are introduced based on the charge distribution along the molecule to represent locations with delocalized negative charges. These latter sites do not interact with each other. COSMO-RS σ-profiles allow for the identification of molecular regions with high electrostatic potential, which can be assumed to function as associative sites in the soft-SAFT modeling.

Consequently, the association scheme of the studied PILs was set based on DFT calculations and the COSMO framework. To this end, Turbomole and COSMOtherm packages were utilized to optimize the molecular geometries of the ions and analyze the resulting structures, respectively. Following previous works,^17,31^ DFT calculations for each studied ion were performed using the COSMO-BP-TZVP parametrization, which involves geometry optimization at the BP86/def-TZVP theoretical level under COSMO solvation conditions. Frequency calculations were conducted to ensure the global energy minimum for each optimized species. The σ-profile for each PIL was then obtained as the sum of the profiles of the ions conforming them.

Figure 1 shows the 3D optimized COSMO surfaces for each ion used in this study. The increase in molecular polarity is depicted by using a color spectrum ranging from blue to red. Blue shades represent areas with a higher positive charge, typically hydrogen donors, while red shades indicate regions with a greater negative charge, usually hydrogen acceptors. Figure 2 presents the σ-profiles of the PILs, which provide valuable information about their structure, including polarity (as indicated by the color bar at the top of the plots) and concentration of specific atoms.

**Figure 1:**
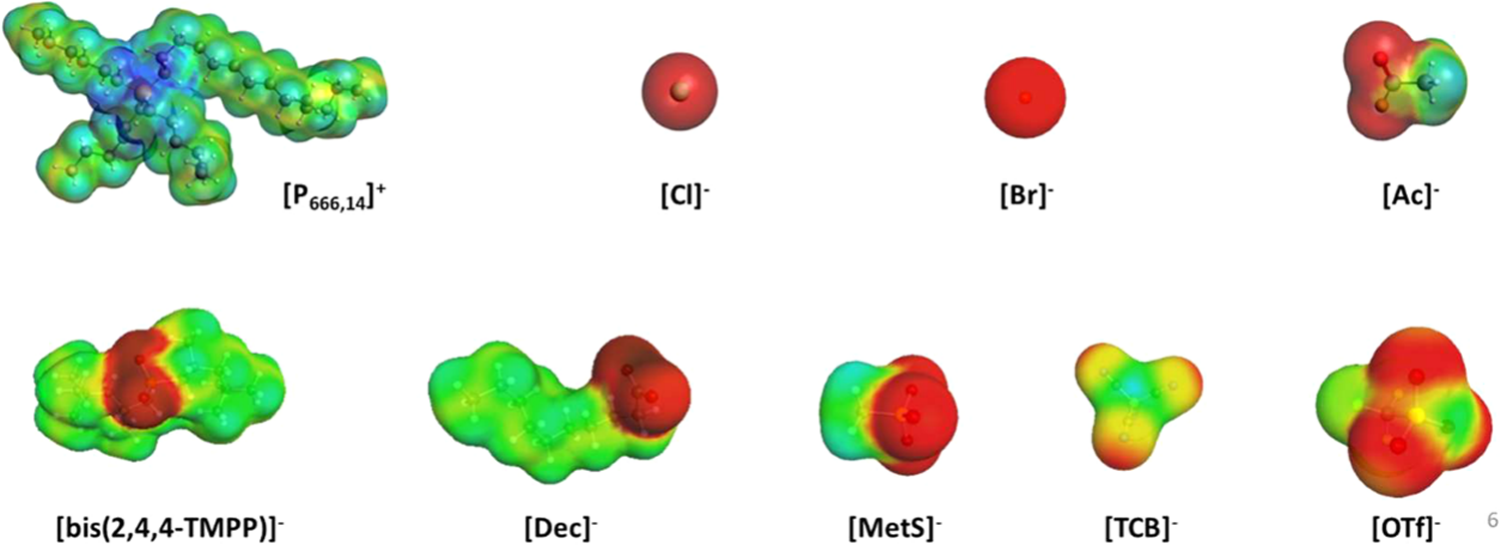
3D geometrically optimized COSMO surfaces of the ions used to form the ionic liquids studied in this work.

**Figure 2:**
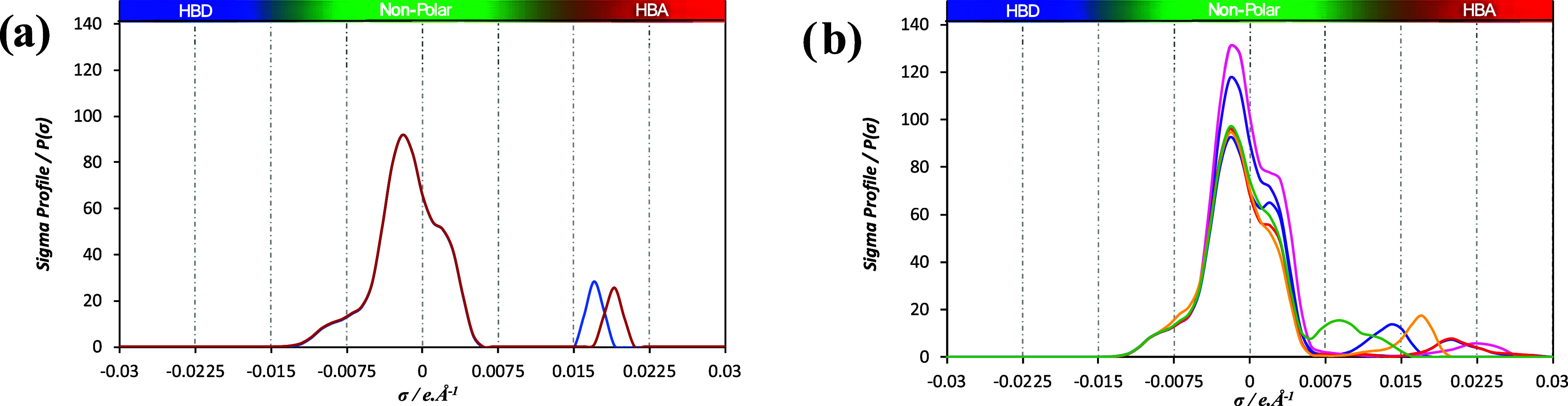
COSMO calculated σ-profiles for the ionic liquids investigated in this study. (a) Blue line [P_666,14_]­[Br] and red line [P_666,14_]­[Cl]; (b) purple line [P_666,14_]­[Dec], red line [P_666,14_]­[Ac], blue line [P_666,14_]­[OTf], yellow line [P_666,14_]­[MetS], pink line [P_666,14_]­[bis­(2,4,4-TMPP)], and green line [P_666,14_]­[TCB].

Following the soft-SAFT molecular model already developed for [P_666,14_]­[Cl],^17^ the model for [P_666,14_]­[Br] incorporates a single dual association site, reflecting the simplicity and localized charge of the anion, a chlorine and a bromide atom, respectively. This is clearly evidenced by the σ-profiles plotted in Figure 2a, where the spherically localized charge of the anion appears as a sharp peak in the positive region, while the spherically localized charge of the cation around the P atom forms a peak in the neutral σ values. For [P_666,14_]­[Ac], the model includes a dual associative site along with an additional negative site to represent charge delocalization between the two oxygen atoms present in the acetate anion.^23^ This is illustrated in the corresponding σ-profile plotted in Figure 2b, which shows a small peak in the positive region. Similarly, [P_66614_]­[Dec] is modeled with a dual associative site and an additional negative site. This methodology aligns with the approach previously used for [P_666,14_]­[bis­(2,4,4-TMPP)].^31^ Finally, [P_666,14_]­[MetS], [P_666,14_]­[OTf], and [P_666,14_]­[TCB] incorporate, in addition to the common dual site used in all ILs, two additional negative sites in their model to account for the charge delocalization in the anion, based on the broader charge distribution along the molecule. The σ-profiles in Figure 2b show substantial peaks in the positive region for these species, which can be assumed to be represented by additional associative sites in the soft-SAFT modeling.

To determine reasonable values for the associative interactions (ε^HB^/*k*_B_ and κ^HB^) and reduce the number of adjustable parameters (so as to avoid parameter degeneracy, which typically happens when working with ILs and other negligible vapor pressure compounds), DFT calculations were carried out. Following previous studies,^17,31^ relative cation–anion interaction energies and equilibrium distances were calculated for the new PILs ([P_666,14_]­[Br], [P_666,14_]­[Dec], [P_666,14_]­[Ac], [P_666,14_]­[OTf], [P_666,14_]­[MetS], and [P_666,14_]­[TCB]), as well as those for [P_666,14_]­[Cl], which had been modeled and validated in previous contributions using soft-SAFT.^17^ The ε^HB^/*k*_B_ and κ^HB^ parameters for these compounds were then scaled to obtain the association parameters for new [P_666,14_]­[X]. This scaling was based on the calculated relative differences in cation–anion DFT interaction energies and equilibrium distances. Both the dual and negatively charged sites were treated with the same parameters, as the electronic density is delocalized across all sites. The remaining dispersive molecular parameters (i.e., *m*, σ, and ε/*k*_B_) were adjusted to experimental single-phase density data under fixed T or P conditions. Further details on the DFT calculations are provided in Section S2 of the Supporting Information.

Moreover, a specific molecular model for CO_2_ is also required. All this information, i.e., the polar coarse-grained soft-SAFT model, including its quadrupole, and the optimized molecular parameters were adopted from previous work.^17^ An important point concerns the extension of the CO_2_ model to mixtures with ILs. While treated as a nonself-associating, yet polar, molecule to describe its pure thermodynamic properties, it is well-known that CO_2_ exhibits specific solvation interactions in the presence of the acetate anion, showing high solubility at low CO_2_ content, which decreases as the CO_2_ content increases. Jog et al.^29^ used *ab initio* calculations to study this chemisorption behavior and concluded that it arises from an acid–base interaction between the carboxylate group and the acidic carbon in the CO_2_ molecule. This phenomenon has been extensively studied in phosphonium-based ILs.^14,15,44,45^ To represent this in soft-SAFT EoS, a cross-association term between CO_2_ and the IL, simulating the interaction, has been included by defining two associative sites for CO_2_ that can interact with the dual site of [P_666,14_]­[Ac] (but cannot interact between them). To adjust the corresponding associative parameters, the CO_2_ solubility data in [P_4444_]­[Ac][Bibr ref15] has been used, provided that no data were available for [Ac]^−^ with the [P_666,14_]^+^ cation. For more details on these adjustments, the reader is referred to Section S5 of the Supporting Information.

The number and nature of the association sites, along with the association parameter values defined based on our analysis, are listed in Table 1.

**Table 1:** Soft-SAFT Molecular Parameters for Phosphonium-Based ILs and Carbon Dioxide Used in This Study

IL	*M*_w_ (g mol^–1^)	*m*	σ_*ii*_ (Å)	ε_*ii*_/*k*_*B*_ (K)	ε_αβ_^HB^/*k*_B_ (K)	κ_αβ_^HB^ (Å^3^)	*N*° sites^a^	error (AAD %)^d^	source
[P_666,14_][TCB]	374.3	9.900	4.051	394.6	2500	2600	1 + 2	1.9 × 10^–2^	this work
[P_666,14_][OTf]	408.5	10.21	3.994	385.9	2900	2300	1 + 2	1.7 × 10^–2^	this work
[P_666,14_][Cl]	519.3	11.23	4.323	386.2	3500	2000	1 + 0		^17^
[P_666,14_][Ac]	542.9	11.34	4.382	386.9	3500	1900	1 + 1	2.50 × 10^–2^	this work
[P_666,14_][Br]	563.8	11.40	4.320	384.0	3300	2100	1 + 0	4.3 × 10^–2^	this work
[P_666,14_][MetS]	578.9	11.60	4.371	367.7	3500	2400	1 + 2	2.3 × 10^–2^	this work
[P_666,14_][Dec]	655.1	12.50	4.519	368.0	3550	2200	1 + 1	3.7 × 10^–2^	this work
[P_666,14_][bis(2,4,4-TMPP)]	773.3	13.52	4.653	379.7	3600	2200	1 + 1		^31^
CO_2_ ^b^	44.01	1.571	3.184	160.2	4430^b^	950^c^			^17^

a The number of sites for ILs is counted as dual sites + negative sites.

b Quadrupole moment parameters for CO_2_: 4.40 × 10^–40^ C/m^2^ and *x*_p_ = 1/3.

c Parameters used to account for cross-association between the IL and CO_2_ when the chemisorption phenomenon is present.

d $\mathrm{AAD}\;\%=\frac{100}{\mathrm{ND}}\times \sum_{i=1}^{\mathrm{ND}}\frac{\left|\rho_{i}^{\mathrm{soft}-\mathrm{SAFT}}-\rho_{i}^{\exp}\right|}{\rho_{i}^{\exp}}$ , where *ND* is the number of data points, ρ_*i*_^exp^ is the experimental density value and ρ_*i*_^soft‑SAFT^ is the density obtained through soft-SAFT EoS at the same conditions.

## 4 Results and Discussion

In this section, we present the application of the soft-SAFT EoS for the screening of PILs capacities for the absorption of CO_2_ in the context of a carbon capture process.

### 4.1 Soft-SAFT Parametrization

After defining the associative sites and parameters, as explained in Section 3, the remaining soft-SAFT EoS parameters were fitted to single-phase liquid density data.^17,23,46^ Measured densities (ρ) for PILs with [Br] and [MetS] anions were obtained from ref 47, whereas, the data for the PILs with [Ac], [Dec], [OTf], and [TCB] anions were taken from ref 48–51 , respectively.

A summary of the molecular parameters found for the different Trihexyltetradecylphosphonium [P_666,14_]^+^ cation-based ILs, along with the number and nature of the considered association sites (i.e., number of hydrogen-bonding interactions allowed) is included in Table 1. [P_66614_]­[Br], [P_666,14_]­[Ac], [P_666,14_]­[MetS], [P_666,14_]­[Dec], [P_666,14_]­[OTf], and [P_666,14_]­[TCB] were adjusted in this work, while the parameters from [P_666,14_]­[Cl], [P_666,14_]­[bis­(2,4,4-TMPP)], and CO_2_ were transferred from refs 17,31 and 52 respectively, and are also included in Table 1 for completeness.

A quick analysis of the parametrization shown in Table 1 reveals that the fitting is accurate, as evidenced by the low AAD % values obtained for all ILs. Figure 3 shows the densities predicted using soft-SAFT for the parametrized PILs at various pressures, compared to experimental data from references.^47–51^ The good agreement between the predicted and experimental densities is confirmed by the plots, with a maximum AAD % of 4.322 × 10^–2^ for [P_666,14_]­[Br], as reported in Table 1.

**Figure 3:**
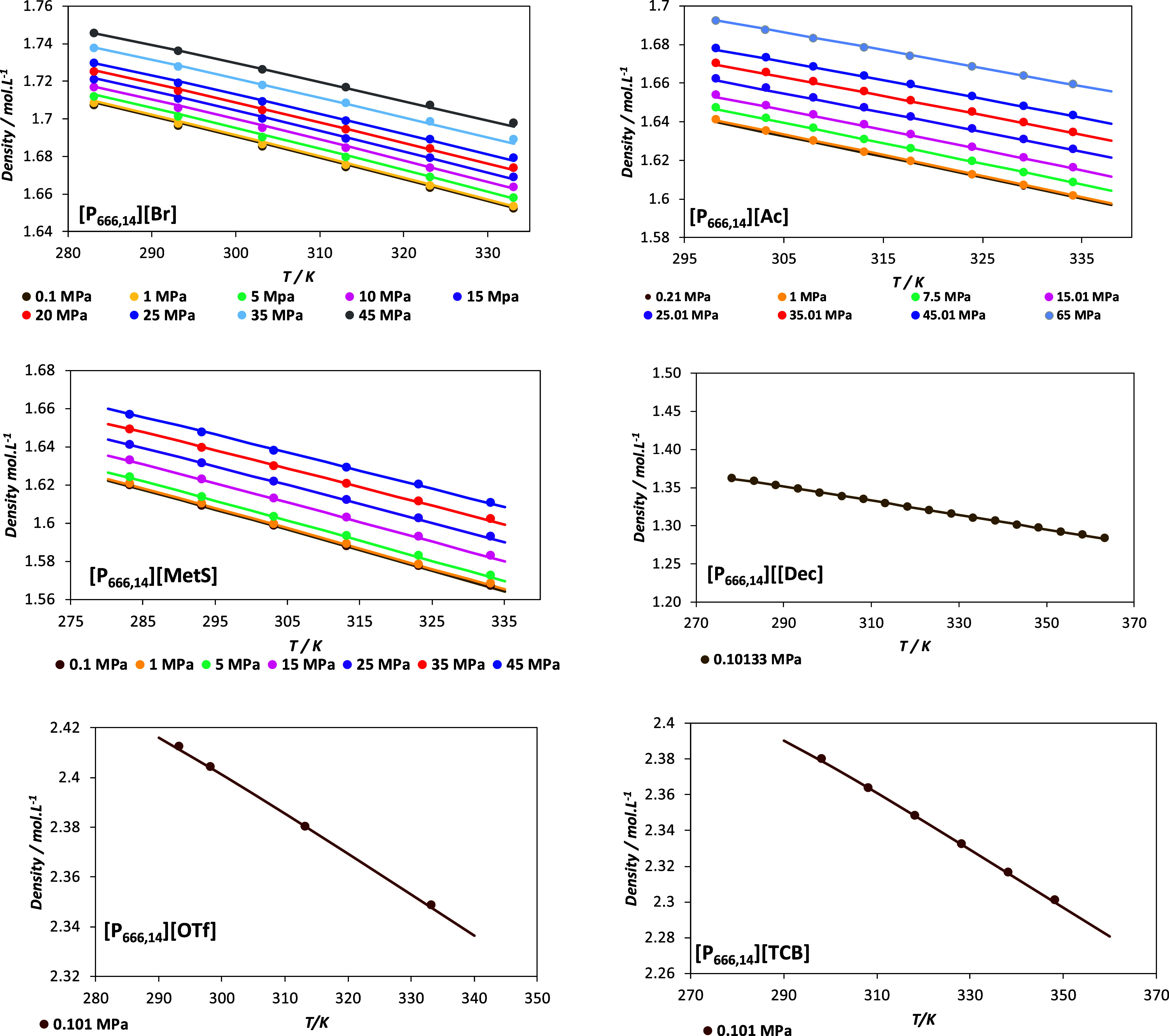
Densities for [P_666,14_]­[Br], [P_666,14_]­[Ac], [P_666,14_]­[MetS], [P_666,14_]­[Dec], [P_666,14_]­[OTf], and, [P_666,14_]­[TCB] at different pressures. Lines represent soft-SAFT calculations with parameters of Table 1 and symbols represent experimental data from.^47–51^

From the parameters obtained, correlations for *m*, *m*σ^3^, and $m\frac{\varepsilon }{k_{B}}$ as a function of the molecular weight of the different ILs can be provided, as previously done for other families, such as alkanes, 1-alkanols,^53^ and imidazolium^54^ ILs. The correlations obtained are listed below, whereas their corresponding plots are shown in Figures S3–S5 of Section S3 in the Supporting Information.

17 $$m=0.009060\times M_{w}+6.461\;R^{2}=0.9932$$

18 $$m\sigma^{3}=1.832\times M_{w}-64.67\;R^{2}=0.9818$$

19 $$m\frac{\varepsilon }{k_{B}}=2.921\times M_{w}+2745\;R^{2}=0.9407$$

### 4.2 Estimation of Transport Properties: Viscosity

As explained in the Section 2, the viscosities of the PILs were calculated by using the FVT treatment combined with soft-SAFT, with the FVT parameters of each molecule fitted to available experimental data. The density values required for the FVT calculations were obtained using the soft-SAFT EoS based on the proposed molecular models. The optimized parameters are listed in Table 2.

**Table 2:** FVT Viscosity Parameters Fitted in This Work for the PILs Used in This Study

IL	$\alpha \left(\frac{{J\,m}^{3}}{\mathrm{mol}\,\mathrm{kg}}\right)$	β	*L* (Å)	AAD %^a^
[P_666,14_][Cl]	696.9	2.850 × 10^–3^	3.734 × 10^–2^	5.0
[P_666,14_][Ac]	476.0	3.432 × 10^–3^	1.474 × 10^–1^	2.9
[P_666,14_][Br]	660.7	2.808 × 10^–3^	3.618 × 10^–2^	4.9
[P_666,14_][MetS]	530.8	3.909 × 10^–3^	3.434 × 10^–2^	5.6
[P_666,14_][Dec]	502.6	4.304 × 10^–3^	1.888 × 10^–1^	5.3
[P_666,14_][bis(2,4,4-TMPP)]	513.49	3.476 × 10^–3^	3.314 × 10^–1^	5.5

a $\mathrm{AAD}\;\%=\frac{100}{\mathrm{ND}}\times \sum_{i=1}^{\mathrm{ND}}\frac{\left|\mu_{i}^{\mathrm{FVT}}-\mu_{i}^{\exp}\right|}{\mu_{i}^{\exp}}$ , where *ND* is the number of data points, μ_*i*_^exp^ is the experimental viscosity value, and μ_*i*_^FVT^ is the viscosity obtained through the FVT model at the same conditions.

Figure 4 illustrates the model’s performance in describing the viscosities of pure PILs, showing strong agreement between the experimental viscosities^49,55,56^ and those calculated with soft-SAFT + FVT (see the corresponding AAD % in Table 2). The largest deviations are observed for [P_666,14_]­[MetS] at low temperatures. A slight decline in accuracy is generally noted at the highest viscosity values, where experimental data also tend to have greater uncertainty.

**Figure 4:**
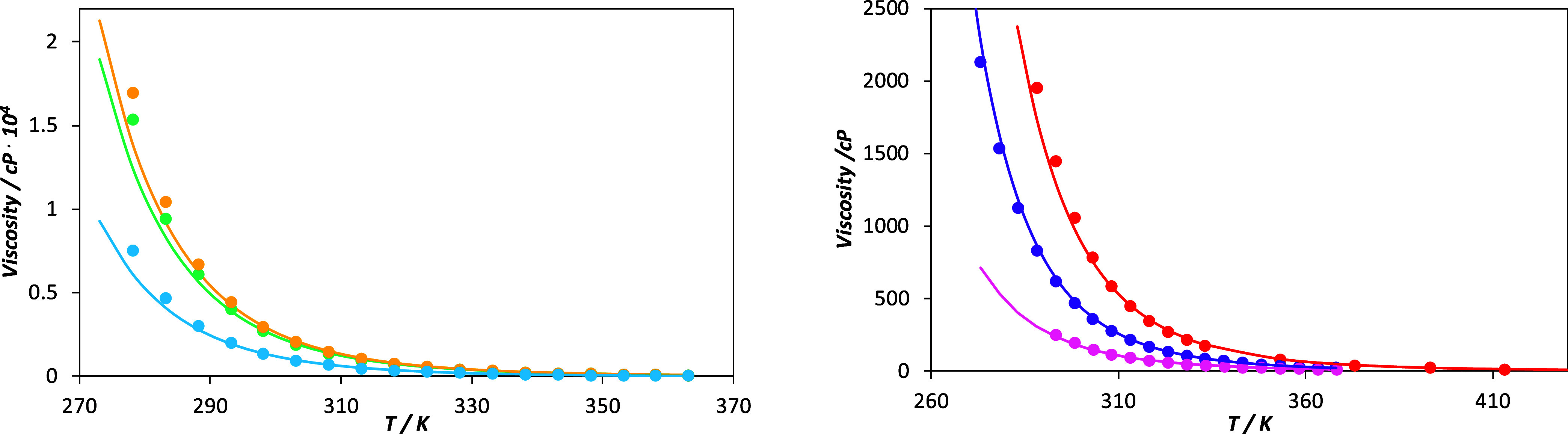
Viscosity (η) – temperature (*T*) diagram, at atmospheric pressure, modeled using FVT parameters shown in Table 2 for: blue Circle [P_666,14_]­[MetS], green circle [P_666,14_]­[Cl], yellow circle [P_666,14_]­[Br], pink circle [P_666,14_]­[Ac], purple circle [P_666,14_]­[Dec], and red circle [P_666,14_]­[bis­(2,4,4-TMPP)]. In all figures, symbols correspond to experimental data^49,55,56^ and lines to soft-SAFT calculations (with parameters from Table 2).

Examining the fitted parameter values from Table 2, it becomes evident that the energy barrier parameter (α) correlates with the cohesive energy of the ionic liquid. ILs with more cohesive single-atom anions exhibit higher viscosities and, correspondingly, higher α values. Conversely, the acetate anion, associated with lower viscosity, presents the lowest energy barrier. Regarding the free-volume parameter β, lower values are observed for chloride- and bromide-based ILs, as the smaller anions allow for more efficient molecular packing and reduced free volume. As bulkier anions are considered—such as decanoate—β increases significantly due to steric hindrance introduced by the long alkyl chain.

The interpretation of the *L* parameter is less direct, as it embodies both molecular size and frictional effects (*Lv*/ϕ). Nonetheless, we observe that ILs with lower viscosity (see Figure 4b) tend to exhibit *L* values approximately 1 order of magnitude higher than those of higher-viscosity ILs (Figure 4a). This trend can likely be attributed to reduced friction coefficients in lower-viscosity systems.

### 4.3 Solubility of CO_2_ in PILs

Once the molecular models have been parametrized and the key thermophysical properties (density and viscosity) for the pure PILs have been adequately described, their CO_2_ absorption capacity for potential carbon capture applications has been evaluated. When available, experimental data for CO_2_ absorption in [P_666,14_]­[Br],^57^ [P_666,14_]­[MetS],^58^ and [P_666,14_]­[bis­(2,4,4-TMPP)][Bibr ref57] are used to fit the soft-SAFT IL–CO_2_ binary parameters. If necessary, typically an intermediate isotherm is employed to fit the binary parameters, which are then applied to other temperatures in a predictive manner, making the parameter temperature-independent. Additionally, the previously adjusted ξ_IL–CO_2__ parameter for the [P_666,14_]­[Cl]–CO_2_ binary system has been transferred to this work.^17^ Table 3 summarizes all of the parameters used in the binary systems. The ξ_IL–CO_2__ values obtained are lower than unity, indicating that the Lorentz rule overestimates the IL–CO_2_ energy interactions and the solubility of CO_2_ in all cases. It is worth noting that the binary size parameter, η_*ij*_, was set to unity for all systems studied in this work, hence reducing eq 2 to the classical Lorentz rule.

**Table 3:** Soft-SAFT Binary Parameters for PIL–CO_2_ Pairs Used in This Study

IL	ξ_IL–CO_2__	comment
[P_666,14_][TCB]	0.90	transferred, more details in the text
[P_666,14_][OTf]	0.90	transferred, more details in the text
[P_666,14_][Cl]	0.94	fitted to data from refs 59 by^17^
[P_666,14_][Ac]	0.90	transferred, more details in the text
[P_666,14_][Br]	0.91	fitted to data from ref 57
[P_666,14_][MetS]	0.895	fitted to data from ref 58
[P_666,14_][Dec]	0.90	transferred, more details in the text
[P_666,14_][bis(2,4,4-TMPP)]	0.88	fitted to data from ref 57

The accuracy of the description of the CO_2_ solubility is shown in Figure 5 for the three previous PILs with [Br], [MetS], and [bis­(2,4,4-TMPP)] anions. Overall, very good agreement is obtained between the experimental data and the soft-SAFT modeling, regardless of the isotherm studied, highlighting the capacity of the model to predict the behavior at different temperatures using a temperature-independent binary parameter.

**Figure 5:**
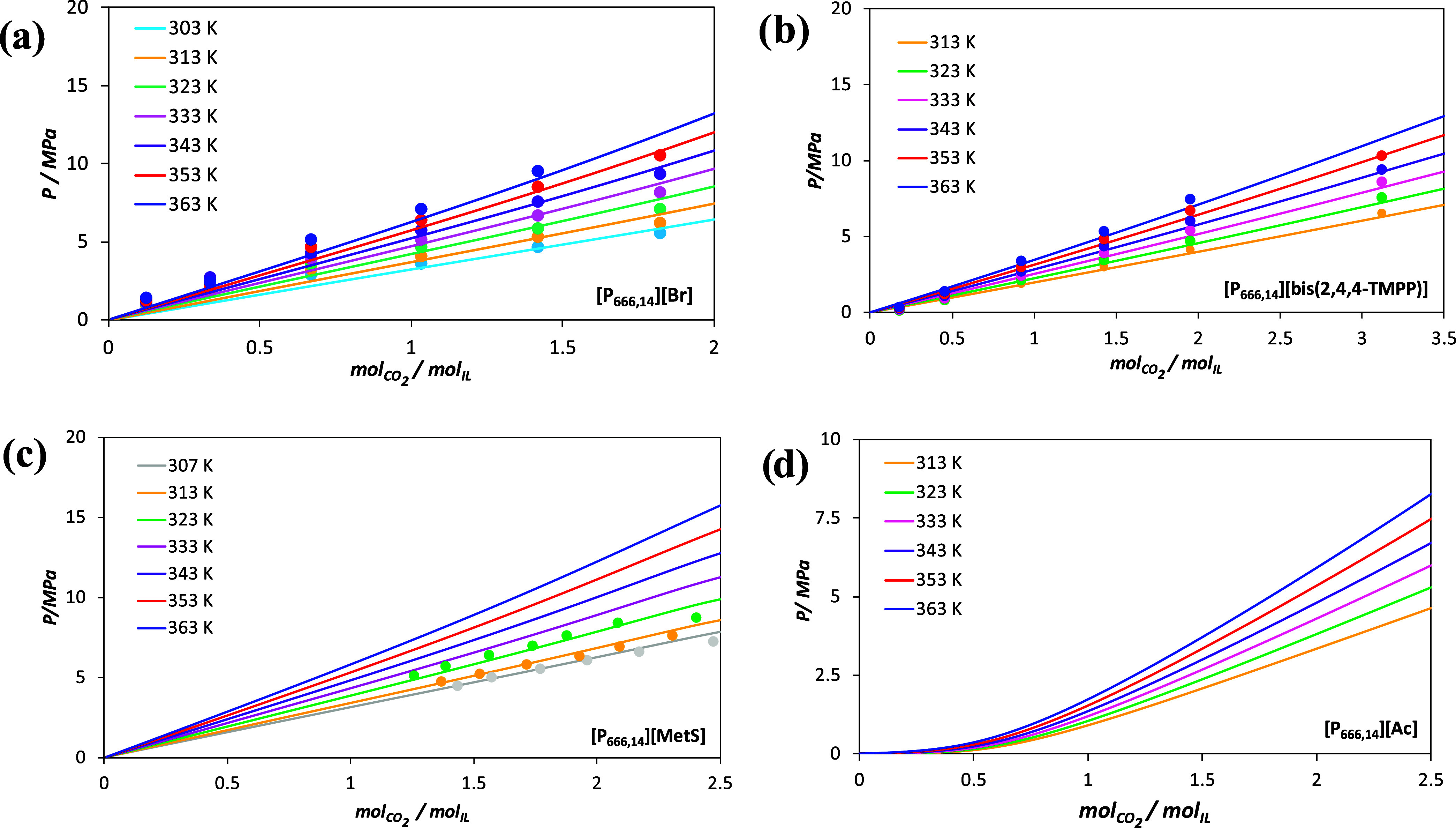
Carbon dioxide solubility in (a) [P_666,14_]­[Br], (b) [P_666,14_]­[bis­(2,4,4-TMPP)], (c) [P_666,14_]­[MetS], and (d) [P_666,14_]­[Ac] at different temperatures. Lines correspond to soft-SAFT calculations (lines) with binary parameters of Table 3, while symbols represent experimental data from refs 57,58 .^57,58^

An important remark refers to the CO_2_ solubility in [P_666,14_]­[OTf], [P_666,14_]­[TCB], and [P_666,14_]­[Dec], where no experimental data are available. However, as these ILs exhibit the same physical absorption mechanism as the previously adjusted systems, we have calculated the average value of the energy binary parameters fitted for [P_666,14_]­[Br], [P_666,14_]­[MetS], [P_666,14_]­[bis­(2,4,4-TMPP)], and [P_666,14_]­[Cl]. This average value was then used to predict the CO_2_ solubility in these ILs. The obtained ξ_IL–CO_2__ values are also included in Table 3, and the predictions are shown in Figure S6 in Section S4 of the Supporting Information.

The case of [P_666,14_]­[Ac] is somewhat different. While CO_2_ solubility data are also not available, its absorption mechanism differs from that of the previous analyzed ILs, and a direct transfer of the ξ_IL–CO_2__ binary parameter will not provide a reliable picture of the system. In this particular case, the estimation of the binary interactions has been based on the experimental data for CO_2_ absorption in [P_4444_]­[Ac].^15^ First, a molecular model for pure [P_4444_]­[Ac] has been developed, as explained in the Section 4.1, to later adjust the ξ_IL–CO_2__ parameter using the binary data. The fitting results are very satisfactory, as shown in Figure S7 and Table S2. Next, the ξ_[P_4444_][Ac]–CO_2__ parameter has been transferred to the [P_666,14_]­[Ac] + CO_2_ system, assuming similar IL–CO_2_ interactions since both ILs are phosphonium-based, share the same anion, and, more importantly, exhibit the same chemisorption mechanism. The CO_2_ solubility predictions for [P_666,14_]­[Ac] at different temperatures are shown in Figure 5d.

### 4.5 Assessment of ILs Performance for CO_2_ Capture through KPIs

The soft-SAFT molecular models presented in the previous sections are now applied predictively to evaluate three key performance indicators for the effectiveness of the solvents in CO_2_ capture: cyclic working capacity, enthalpy of absorption, and diffusivity. The study is based on thermodynamic equilibrium calculations and assumes pure CO_2_ as the flue gas. As a result, the reported KPIs are intended for prescreening purposes only, as solvent selectivity and kinetic effects are not directly accounted for. However, the CO_2_ solubility, viscosity, and diffusivity are evaluated to provide qualitative insights into potential mass transfer limitations.

#### 4.5.1 Solvent Cycling Working Capacity

The cyclic working capacity for each IL has been assessed through eq 7 considering three different operation options for the system: (a) absorption at high pressures and regeneration by pressure swing (PS); (b) absorption at atmospheric pressures and desorption by temperature swing (TS); and (c) absorption at high pressures and desorption combining pressure and temperature swing (TPS).

For the assessment of the operation of the regeneration by pressure swing, the absorption capacity of the solvents has been evaluated at 313 K under two CO_2_ partial pressures, 1 and 2.5 MPa, to analyze the pressure effect on the process. Desorption conditions are fixed at the same temperature and vacuum conditions (0.01 MPa). The required amount of solvent per ton of CO_2_ absorbed is calculated based on the results obtained with soft-SAFT at the working temperature of 313 K, as shown in Figure 6.

**Figure 6:**
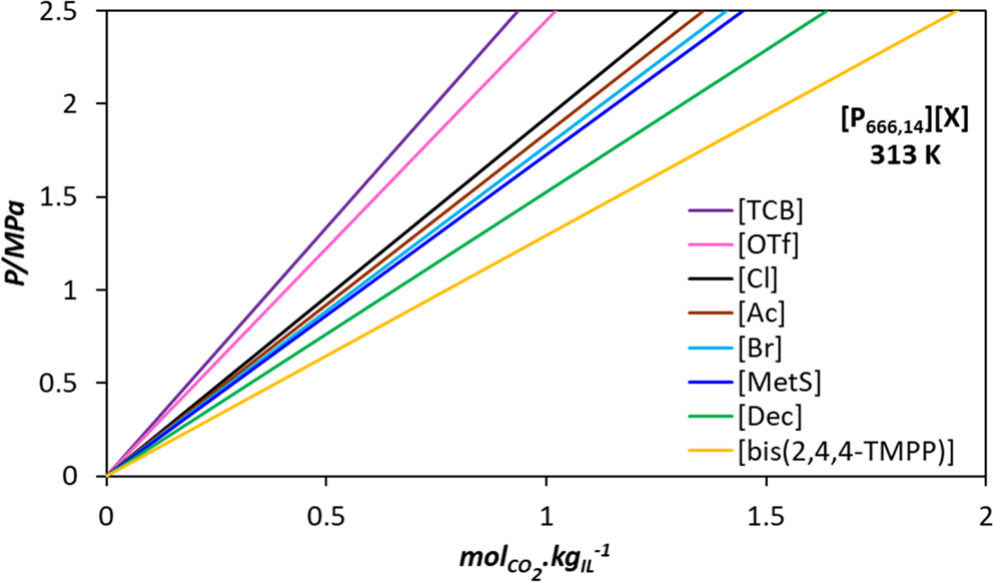
CO_2_ loading at 313 K for all the PILs studied in this work as a function of pressure, from 0.0001 to 2.5 MPa, predicted from soft-SAFT (lines) with parameters from Tables 1 and 3.

For [P_666,14_]^+^ based ILs, the CO_2_ solubility increases in the following order [Ac] > [bis­(2,4,4-TMPP)] > [Dec] > [Cl] > [MetS] ≈ [Br] > [TCB] ≈ [OTf]. The highest predicted CO_2_ loading capacity, 1.04 mol CO_2_ per mol of IL, at 1 MPa and 313 K, is observed for [P_666,14_]­[Ac] (see Figure S6 in Section S4 of the Supporting Information). This result was expected since [P_666,14_]­[Ac] operates via a chemical absorption mechanism, unlike the other ILs, which act as physical solvents. Following [P_666,14_]­[Ac], [P_666,14_]­[bis­(2,4,4-TMPP)] shows the second highest predicted loading capacity of 0.51 mol CO_2_ per mol of IL under the same conditions.

The results for the cyclic working capacity and solvent required per ton of CO_2_ absorbed are presented in Figure 7. Increasing the absorption pressure from 1 to 2.5 MPa results in approximately a 60% increase in working capacity for most solvents, except for [P_666,14_]­[Ac], which shows a smaller increase of 43%. The highest working capacities are observed for [P_666,14_]­[Ac] and [P_666,14_]­[bis­(2,4,4-TMPP)], with values of 1.45 and 1.26 mol of CO_2_ per mol of IL at 2.5 MPa, and 0.82 and 0.51 mol of CO_2_ per mol of IL at 1 MPa, respectively. Furthermore, a reduction in cyclic working capacity between 2.5 and 9.2% is observed for the physical solvents and up to 31.9% for [P_666,14_]­[Ac], when comparing processes in which absorption occurs at 1 MPa and desorption is carried out at 0.1 MPa instead of 0.01 MPa.

**Figure 7:**
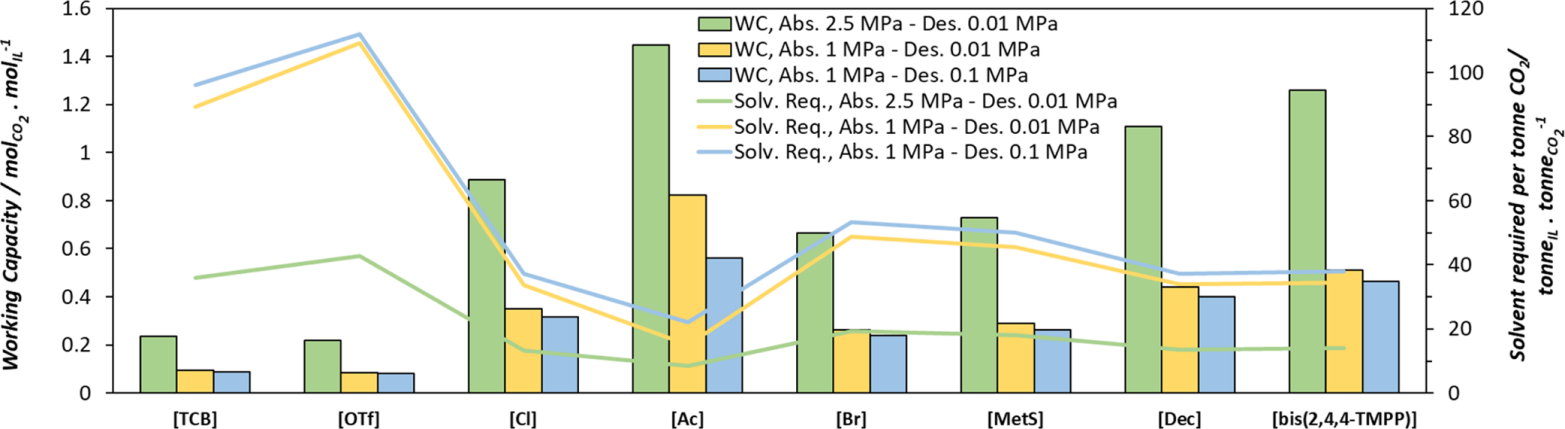
Solvent cyclic working capacity at 313 K and solvent required per tonne of CO_2_ absorbed for the studied PILs for pressure swing CO_2_ separation processes. Green: absorption at 2.5 MPa/desorption at 0.01 MPa; orange: absorption at 1 MPa/desorption at 0.01; and blue: absorption at 1 MPa/desorption at 0.1 MPa.

Regarding solvent cyclic capacity, an inverse relationship is observed when comparing at the same desorption pressure of 0.01 MPa: higher operating pressures and cyclic working capacities result in lower solvent requirements. [P_666,14_]­[Ac] has the lowest solvent consumption, requiring 8.52 or 14.99 tonnes of IL per tonne of CO_2_ captured at 2.5 and 1 MPa, respectively. In comparison, capturing 1 ton of CO_2_ with [P_666,14_]­[bis­(2,4,4-TMPP)] requires 13.94 or 34.48 tons of solvent under the same conditions. [P_666,14_]­[Dec], [P_666,14_]­[Cl], exhibit similar solvent requirement to [P_666,14_]­[bis­(2,4,4-TMPP)]. Other ILs including [P_666,14_]­[MetS], [P_666,14_]­[Br], [TCB], and [OTf] showed higher solvent requirements compared to those of [P_666,14_]­[Ac]. Additionally, when comparing the pressure swing process operating at 1 MPa of CO_2_ partial pressure during absorption and varying pressures for the desorption—0.1 and 0.01 MPa—an increase in the mass of solvent required to remove a tonne of CO_2_ between 2.5 and 10.2% is observed for the physical solvents, increasing up to 46.8% for [P_666,14_]­[Ac].

For the assessment of the operation of the regeneration by a temperature swing, we assumed CO_2_ capture at 313 K and 0.1 MPa of CO_2_ pressure and the desorption operating in a column at 373 K and the same pressure. In Figure 8, we continue observing the working capacity tendencies already explained for the studied PILs, with [P_666,14_]­[Ac] and [P_666,14_]­[bis­(2,4,4-TMPP)] showing the highest WC of 0.45 and 0.05 mol of CO_2_ per mol of IL, respectively. Even though the operation by temperature swing seems to be the less convenient for all the analyzed PILs, this effect is more pronounced for the physical solvents, for which the pressured operation should be considered when evaluating the working capacity and solvent requirements. For the particular case of [P_666,14_]­[Ac], the operation by a temperature swing also put down its sorption capacity, but to a lower extent.

**Figure 8:**
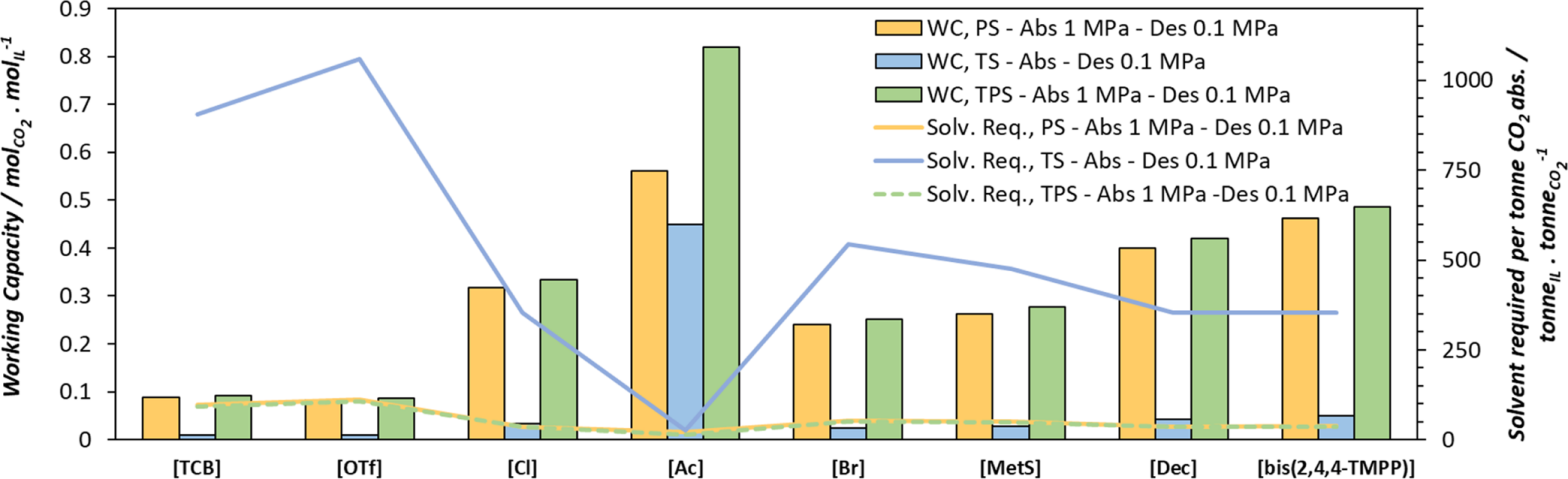
Solvent cyclic working capacity and solvent required per tonne of CO_2_ absorbed for the studied PILs. All processes operate absorption at 313 K. Orange: pressure swing; blue: temperature swing; green: temperature and pressure swing. Detailed conditions are summarized in Table 4.

Finally, a process combining pressure and temperature swing for desorption was assessed, assuming CO_2_ absorption at 313 K and 1 MPa, and IL regeneration at 0.1 MPa and 373 K in the desorber. This was done to enable comparison to the previously analyzed Ps and TS processes. Figure 8 presents the comparison of the working capacity and the solvent required per tonne of CO_2_ absorbed for the three process configurations, according to the conditions listed in Table 4.

**Table 4:** Operating Conditions for the Processes Assessed in Figure 8

process	absorption conditions	desorption conditions
pressure swing (PS)	313 K, 1 MPa	313 K, 0.1 MPa
temperature swing (TS)	313 K, 0.1 MPa	373 K, 0.1 MPa
temperature and pressure swing (TPS)	313 K, 1 MPa	373 K, 0.1 MPa

Figure 8 shows that, although the working capacity of the solvents is significantly reduced under the TS operation scheme compared to PS, the TPS process exhibits a similar working capacity when comparing physical solvents. Apparently, the effect of the temperature in the desorption step of a TPS process is limited, reinforcing the importance of PS during desorption when using physical solvents. In the case of [P_666,14_]­[Ac], the highest working capacity is achieved under TPS operation, with a low solvent requirement. This is mainly due to the increase in the solvent loading when the absorption steepness is performed at high pressure.

#### 4.5.2 Energy of Regeneration

The energy required for the regeneration of the studied solvents was assessed using eq 12 at 313 K and across a pressure range of 0.01 to 1 MPa, as shown in Figure 9. For all the studied PILs, increasing the pressure reduces the energy required for desorption but consequently decreases the amount of CO_2_ recovered.

**Figure 9:**
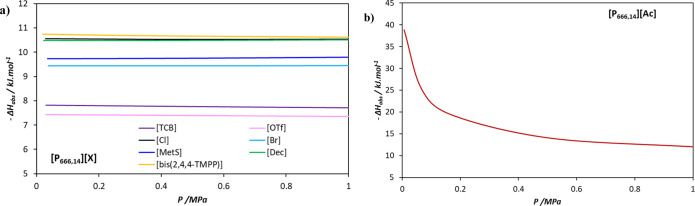
Enthalpy of desorption at 313 K as a function of pressure in the studied PILs evaluated with soft-SAFT EoS.

Figure 9a shows the enthalpy of desorption for all physical solvents. Among these and across the analyzed pressure range, [P_666,14_]­[bis­(2,4,4-TMPP)] exhibits the highest values of −Δ*H*_abs_ (∼10.7 kJ mol^–1^), while [P_666,14_]­[OTf] has the lowest value (∼7.4 kJ mol^–1^). The obtained desorption enthalpies are consistent with those reported for ILs, which act as physical solvents.^60^ In contrast, Figure 9b shows the enthalpy of desorption for [P_666,14_]­[Ac], which is significantly higher across the pressure range, reaching approximately 38 kJ mol^–1^ at 0.01 MPa, as a consequence of the difficulty to revert the chemisorption occurred. However, even in this latter case, the enthalpy of desorption remains lower than that of MEA or MDEA 30 wt % aqueous solutions at 313 K (85.13 and 52.51 kJ mol^–1^, respectively, as reported by ref 61). Furthermore, all the physical solvents studied also present a lower enthalpy of desorption than other commercially used physical solvents, such as Selexol (−Δ*H*_abs_ = 14.3 kJ mol^–1^) and Rectisol (−Δ*H*_abs_ = 13 kJ mol^–1^).^60^ This could favor the economic viability of the process, provided that these alternative solvents exhibit good absorption capacities.

Additionally, the energy consumption for the separation of one tonne of CO_2_ was assessed for the three processes with the absorption/desorption conditions described in Table 4. [P_666,14_]­[TCB] and [P_666,14_]­[OTf] ionic liquids have been excluded from this analysis since they had shown a very low working capacity under all of the process configurations (see Figure 8), and their isobaric heat capacities are not available in the open literature to estimate the sensible enthalpy.

Figure 10 clearly shows that the thermal energy required for the TPS process, obtained through eq 12, is much higher than that required for the PS-driven desorption process. In the PS process, the energy consumption is basically due to the desorption enthalpy, obtained by eq 14, whereas in the TPS process, the overall energy consumption is mainly due to the sensible heat, which is related to the isobaric heat capacity and the working capacity of the IL. The isobaric heat capacities used in this work are reported in Table S3 in the Supporting Information. For comparative purposes, the heat capacities at 313 K were estimated and are reported in Table S3. The values range between 634.0 J mol^–1^ K^–1^, for [P_666,14_]­[MetS], to 1665.3 J mol^–1^ K^–1^, for [P_666,14_]­[bis-2,4,4-TMPP]. In the particular case of [P_666,14_]­[Br] a high *C*_p_ value (1067.8 J mol^–1^ K^–1^) combined with a low working capacity for the TPS process, as observed in Figure 8, provides a high energy consumption. This effect of high heat capacity and low working capacity is even more pronounced in the case of the TS process, as observed from Figure S8 in the Supporting Information. Although not evaluated in this study, it is worth noting that heat integration in the proposed processes could further reduce energy consumption.^62^ Additionally, compression work should be considered if the gas is pressurized before entering the column, as well as the energy consumption of the vacuum system if desorption is carried out at subatmospheric pressures. These energy requirements associated with pressure changes were not assessed in this work.

**Figure 10:**
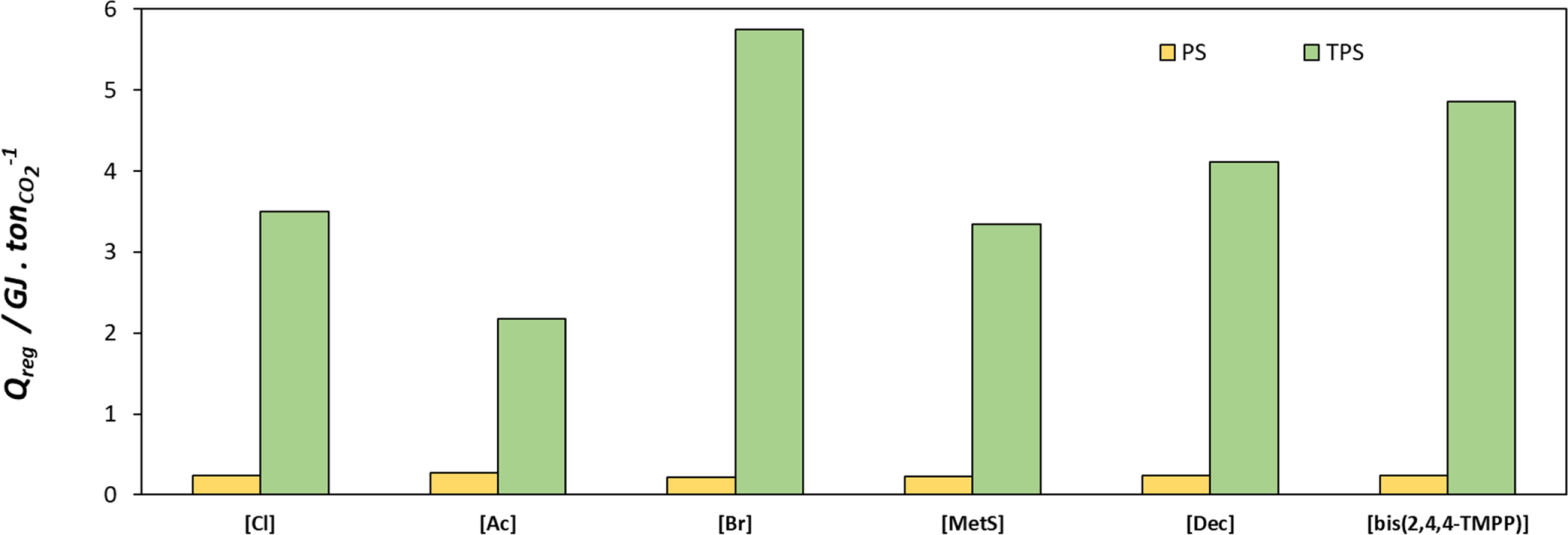
Energy consumption for a PS and a TPS CO_2_ separation process with the conditions detailed in Table 4. Orange: pressure swing; green: temperature and pressure swing.

#### 4.5.3 Mass Transfer

The design of the CO_2_ capture units is significantly influenced by mass transfer effects. Diffusivity and viscosity are key properties that affect mass transfer within the column and have a direct impact on capital cost. Hence, in Figure 10, the diffusivity of CO_2_ in the studied ILs has been estimated using eq 8, assessing the viscosity of the solvent and the molar volume of CO_2_ through soft-SAFT EoS at 313 K and 1 MPa. [P_666,14_]­[OTf] and [P_666,14_]­[TCB] have been excluded from the analysis due to their low predicted CO_2_ absorption capacities, as discussed in Section 4.4.1.

Figure 11 shows the solvent viscosity as a function of the working capacity for the TPS process operating under the conditions reported in Table 4. The sizes of the balls in Figure 11 represent diffusivity. The results indicate higher diffusivity for acetate and decanoate-based ILs, followed by bis­(2,4,4)­TMPP and methylsulfonate-based ILs. This trend is directly related to the viscosity of the solvents, where lower viscosity corresponds to a higher CO_2_ diffusion coefficient. For an optimal solvent, both a high diffusion coefficient and a high working capacity are desirable. Based on these criteria, [P_666,14_]­[Ac], [P_666,14_]­[bis­(2,4,4-TMPP)], and [P_666,14_]­[Dec] demonstrate the best performance, with acetate clearly ahead. It is worth mentioning that CO_2_ absorption tends to increase solvent viscosity, especially at high working capacities, although this effect has not been evaluated in the present study.

**Figure 11:**
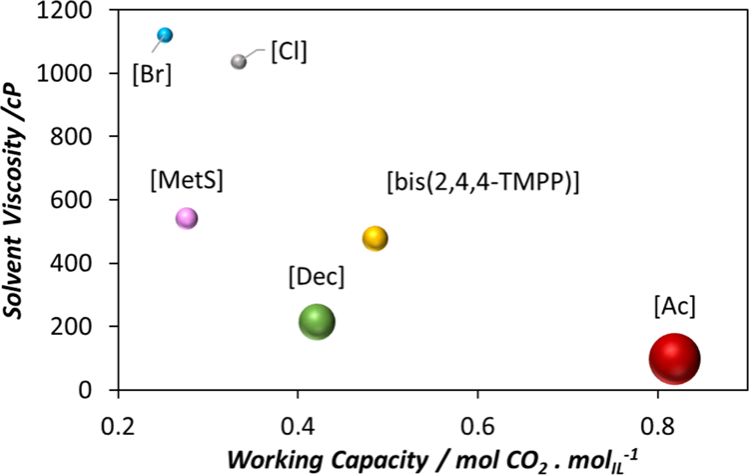
Comparative assessment of PILs performance under TPS operation during the desorption process. Results were obtained using soft-SAFT simulations under the conditions reported in Table 4 for the process. The size of the balls represents the diffusivity of the CO_2_ in each solvent.

Additional comparative assessments for PS processes are presented in Section S7 of the Supporting Information.

#### 4.5.4 Benchmarking of Solvents for CO_2_ Capture

The selection of the optimal solvent for CO_2_ capture requires balancing all KPI’s used in this study. Performance of the process was assessed under different conditions of absorption and desorption to evaluate the effects of the temperature and pressure changes. An ideal solvent should exhibit a high working capacity, high CO_2_ diffusivity, and low energy requirements for desorption.

Among the examined PILs, and under all of the operation modes, [P_666,14_]­[Ac] demonstrated the best performance regarding working capacity and CO_2_ diffusivity.

Alternatively, [P_666,14_]­[bis­(2,4,4-TMPP)] and [P_666,14_]­[Dec] showed comparable enthalpies of desorption among them. However, [P_666,14_]­[bis­(2,4,4-TMPP)] exhibited a higher working capacity and a lower CO_2_ diffusivity than [P_666,14_]­[Dec]. Overall, both solvents seem to perform similarly, with their enthalpies of desorption being more favorable than [P_666,14_]­[Ac] compared to conventional aqueous amine solutions, although at the cost of a lower working capacity.

## 5 Conclusions

In this work, the performance of a variety of phosphonium-based ionic liquids, particularly containing the [P_666,14_]^+^ cation, has been assessed concerning its potential to be used as solvents in CO_2_ capture processes. To that end, new soft-SAFT molecular models have been developed for [P_666,14_]­[MetS], [P_666,14_]­[OTf], [P_666,14_]­[TCB], [P_666,14_]­[Ac], [P_666,14_]­[Br], and also [P_4444_]­[Ac], whereas models for [P_666,14_]­[Cl] and [P_666,14_]­[bis­(2,4,4-TMPP)], and CO_2_ have been transferred from previous contributions. The new coarse-grained models have been constructed with the help of DFT calculation techniques (i.e., TURBOMOLE/COSMO-RS), which allow the identification of regions within the molecules with higher electrostatic potential, the assignment of associative sites, and the estimation of the energy and distance of association. The obtained soft-SAFT models have been validated through the accurate reproduction of the density of ionic liquids, whereas their viscosities have also been obtained by combining soft-SAFT density predictions with the FVT model.

Soft-SAFT EoS has also been used to describe the absorption isotherms of CO_2_ in the studied PILs, adjusting a temperature-independent energy binary parameter when experimental data were available. Alternatively, the binary parameter ξ_IL–CO_2__ was transferred from a system with similar features. Thereafter, following a procedure developed for the screening of solvents for CO_2_ captured,^22^ the models have been used to assess key indicators related to the performance of the carbon capture process which consider the carbon capture capacity, the diffusivity of CO_2_ in the solvent and the energy required for the regeneration of the solvent. [P_666,14_]­[bis­(2,4,4-TMPP)] and [P_666,14_]­[Dec], both physical sorbents, have demonstrated a similar performance, whereas a better working capacity is observed for [P_666,14_]­[Ac] with an energy of regeneration more than twice as high, which is in accordance with its chemical absorption mechanism. Regarding transport properties of the solvents, low viscosity and high diffusivity are encountered for [P_666,14_]­[Dec] and [P_666,14_]­[Ac] under the considered operating conditions.

These findings should be taken with care. It is advisable to validate these results with new experimental measurements, particularly for [P_666,14_]­[Dec] and [P_666,14_]­[Ac], for which CO_2_ solubilities have not been reported in the open literature. Another important aspect for process operation involves reuse of the solvent and its possible degradation. Additionally, it is also worth mentioning that the solvent cost also plays a role in the implementation of industrial application of ILs. However, these guidelines fall beyond the scope of this work. Still, the combination of multiscale simulation approaches, such as the one presented in this work, enables the prescreening of new solvents in a rapid, efficient, and cost-effective manner. In particular, the integration of thermodynamically grounded performance indicators into solvent evaluation represents a novel contribution, offering a powerful tool to accelerate research progress in key areas such as CO_2_ capture technology development.

## Supplementary Material



## Notes

Schematic representation of ion interactions in the studied phosphonium-based ILs; determination of association parameters through DFT calculations; representation of molecular parameter tendencies; additional results of CO_2_ solubility in PILs; energy consumption for the TS process; and additional mass transfer results (PDF)
