# Supplementary material for: Characterizing the Potential of Phosphonium-Based Ionic Liquids for CO2 Capture via Multiscale Modeling
Source: Ind Eng Chem Res. 2025 Sep 2;64(36):17878–91. doi: 10.1021/acs.iecr.5c01361 (PMC12517470; doi:10.1021/acs.iecr.5c01361)
Supplement: Supplementary file 1 [file ie5c01361_si_001.pdf]

Supporting Information

Characterizing the potential of phosphonium-based ionic liquids for CO_2 capture via multiscale modeling

Sabrina Belén Rodriguez-Reartes[1,2,3*], *Fèlix Llovell*[1]

[1]Department of Chemical Engineering. Universitat Rovira i Virgili.

Campus Sescelades, C/ Marcel·lí Domingo, s/n Tarragona, 43007, Spain.

[2]Departamento de Ingeniería Química, Universidad Nacional del Sur (UNS)

Avda. Alem 1253, Bahía Blanca, (8000), Argentina

[3]Planta Piloto de Ingeniería Química – PLAPIQUI (UNS-CONICET)

Camino "La Carrindanga" Km 7, Bahía Blanca, (8000), Argentina

*Corresponding Author: sabrinabelen.rodriguez@urv.cat

Section S1. Schematic representation of ion interactions in the studied phosphonium-based ILs.

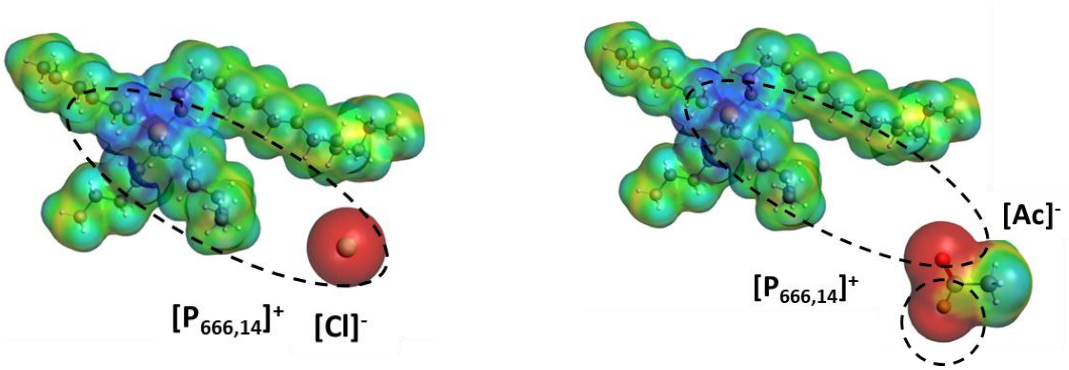

Figure S.1. Schematic representation of the "dual site" interaction between the positively charged region (blue) in the $[P_{666,14}]^+$ cation and the negative region (red) in the anion (black dotted oval). For the $[Cl]^-$ anion, a single negatively charged region appears, whereas $[Ac]^-$ displays two negatively charged regions (in red) distributed around both oxygen atoms.

Section S2. Determination of association parameters through density functional theory (DFT) calculations.

We have computed associating parameters for six PILs which were not previously parametrized by DFT calculations following previous works[1,2]. The association energy ($\varepsilon_{\alpha\beta}^{HB}/k_B$) and volume parameters were determine for [$P_{666,14}$][X] species (i.e., [$P_{666,14}$][Br], [$P_{666,14}$][Dec], [$P_{666,14}$][Ac], [$P_{666,14}$][OTf], [$P_{666,14}$][MetS], and [$P_{666,14}$][TCB]), using shorter alkyl chain lengths in the cation, [P_{3333}]$^+$, to reduce the computational time, and [P_{3333}][Cl], as a reference. The B3LYP/def2-TZVP theoretical level was used in all cases. Dispersion corrections were added via the Grimme's D3 refinement [3]. Given its existing parameters [1], [P_{3333}][Cl] PIL was established as the reference system for the comparisons between interaction energies and distance between both ions. As an example, optimized structures for [P_{3333}][Cl], [P_{3333}][Br] and [P_{3333}][Ac],are presented in Figure S2.

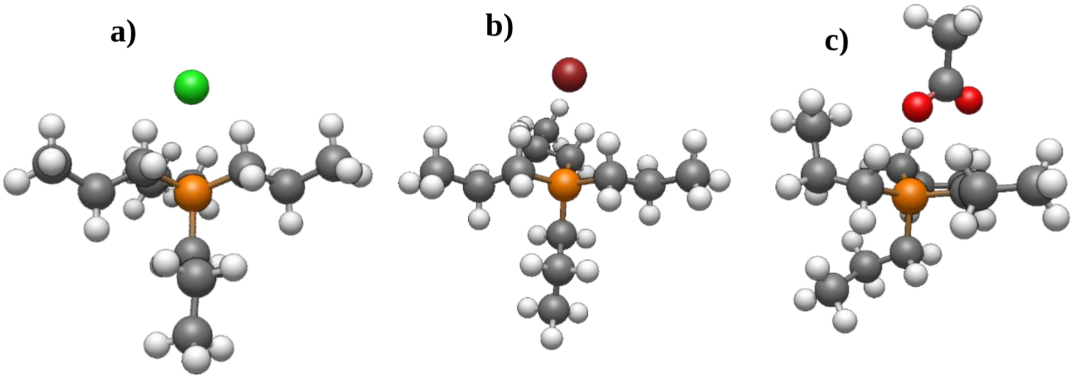

Figure S.2. Optimized structures for [P_{3333}][Cl] (a), [P_{3333}][Br] (b) and [P_{3333}][Ac] (c)

Once the minimum-energy structures were obtained for each PIL, interaction energies and cation-anion distance were computed to obtain the association energy and volume of each species. Assuming that the available association parameters for [P_{66614}][Cl] are equivalent to the ones for [P_{3333}][Cl], a linear scaling of these parameters based on the DFT results allowed

to estimate values for each new PIL. To handle the basis set superposition error, interaction energies, ΔE, were obtained considering the counterpoise correction[4,5] as:

$$\Delta E = E_{IL}^{IL}(IL) - \mathrm{E}_{C}^{C}(C) - \mathrm{E}_{A}^{A}(A) - [E_{C}^{IL}(IL) - E_{C}^{IL}(C) + E_{A}^{IL}(IL) - E_{A}^{IL}(A)] \quad \text{(S1)}$$

where $E_X^Y(Z)$ represents the single-point energy of species X, evaluated at the optimized geometry Y and using the Z basis set. IL, C and A stand for ionic liquid, cation and anion, respectively. The cation-anion distance, r_{CA}, was defined as the distance between the phosphorus atom in the $[P_{3333}]^+$ cation and the center of mass of the functional group in the anion for each case. Results are presented in Table S.1.

Table S1. Interaction energies, ΔE, cation-anion distance, r_{CA}, and associating parameters for all the considered PILs.

**Compound**	ΔE **[kcal·mol⁻¹]**	r_{CA} **[Å]**	ε^{AB}/k_B **[K]**	κ^{AB} **[Å³]**
$[P_{3333}][Cl]$	-93.426	3.374	3500	2000
$[P_{3333}][TCB]$	-67.114	4.367	2500	2600
$[P_{3333}][OTf]$	-77.632	3.905	2900	2300
$[P_{3333}][Ac]$	-94.328	3.184	3500	1900
$[P_{3333}][Br]$	-88.649	3.613	3300	2100
$[P_{3333}][MetS]$	-94.374	4.101	3500	2400
$[P_{3333}][Dec]$	-95.057	3.816	3550	2200

Section S3. Representation of molecular parameter tendencies.

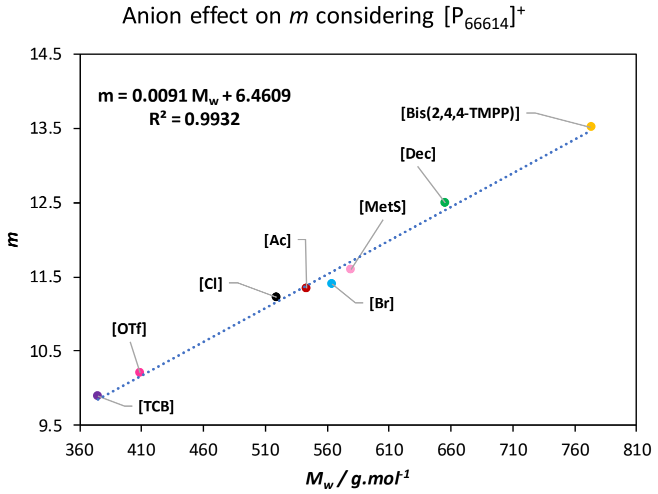

Figure S.3. Tendency of molecular parameter m as a function of the molecular weights (M_w) for the studied PILs evidencing the anion effect on the m parameter over the $[P_{666,14}]^+$IL family.

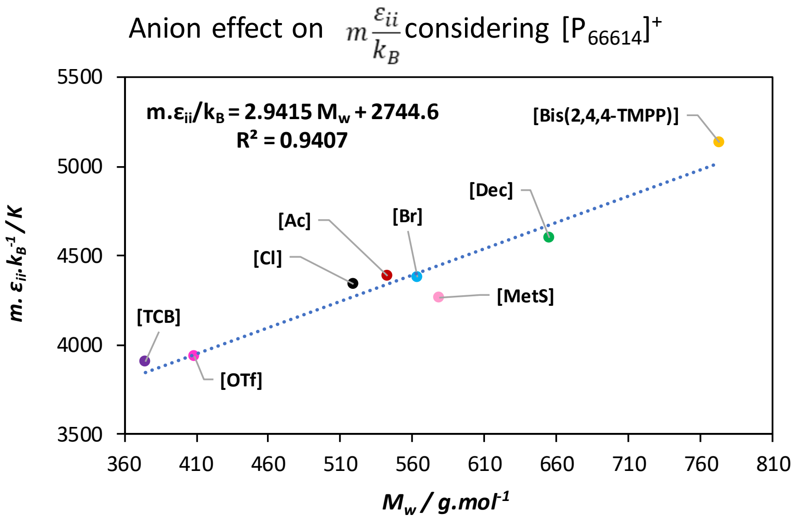

Figure S.4. Tendency of $m\frac{\varepsilon}{k_B}$ as a function of the molecular weights (M_w) for the studied PILs.

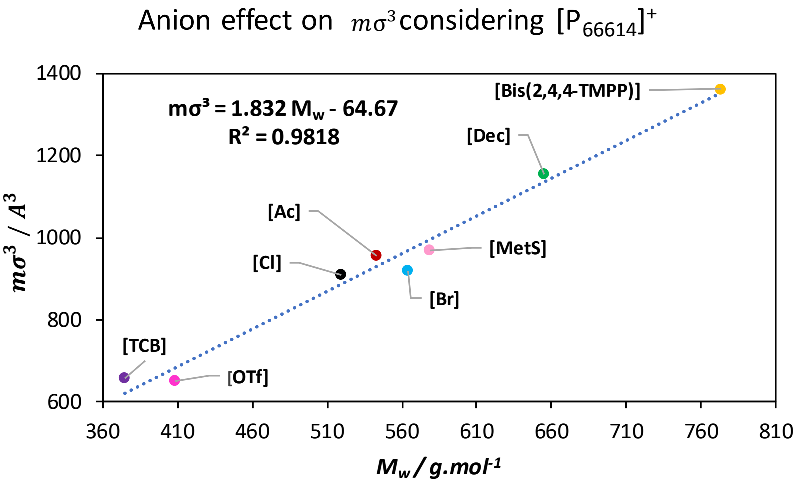

Figure S.5 - Tendency of $m\sigma^3$ as a fucntion of the molecular weights (M_w) for the studied PILs**.**

Section S4. Additional results of CO_2 solubility in PILs.

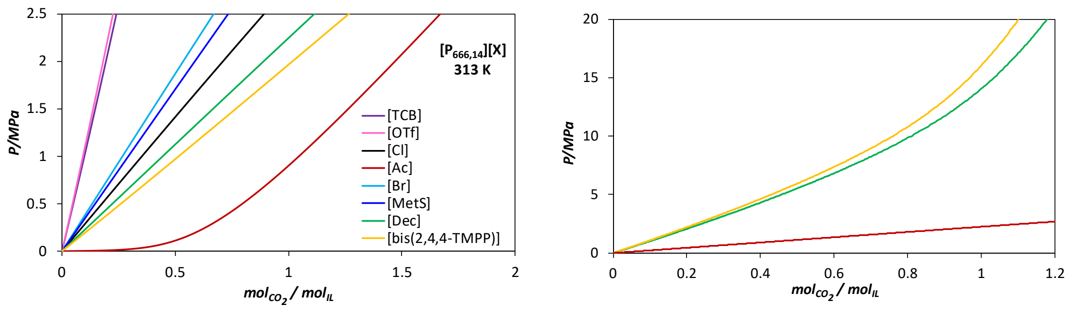

Figure S.6 - Predicted CO_2 absorption at 313 K, (plot on the left): for all the PILs studied in this work as a function of pressure, from 0.0001 to 2.5 MPa, (plot on the right): for — [$P_{666,14}$][OTf], — [$P_{666,14}$][TCB], and — [$P_{666,14}$][Dec] from 0.0001 to 20 MPa. Lines correspond to soft-SAFT calculations with pure compound parameters of Table 1 and binary parameters of Table 3.

Section S5. soft-SAFT molecular model for pure [P_{4444}][Ac]

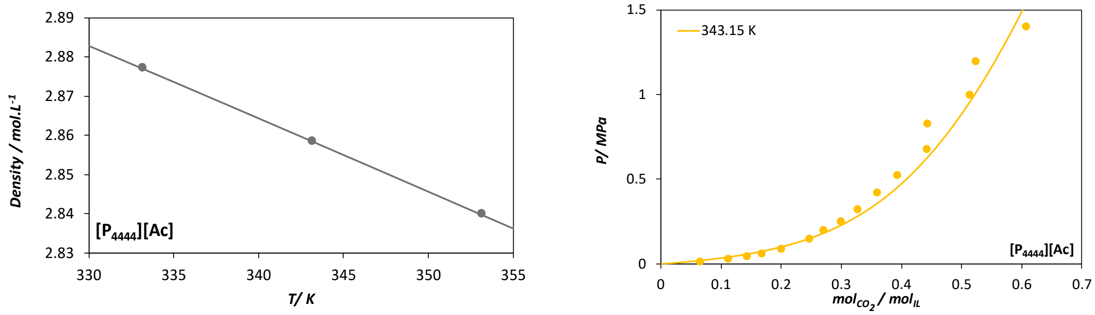

Figure S.7 – Pure [P_{4444}][Ac] density (left) and predicted carbon dioxide absorption at 343.15 K for [P_{4444}][Ac](right). Lines correspond to soft-SAFT calculations with parameters of Tables S2 and Table 1, and binary parameters $\eta_{IL-CO_2} = 1$ and ξ_{IL-CO_2}=0.9. Symbols represent experimental data from[6].

Table S2. soft-SAFT molecular parameters for [P_{4444}][Ac] used in this study.

IL	Mw (g·mol^{-1})	m	σ_{ii} (Å)	ε_{ii}/k_B (K)	$\varepsilon_{\alpha\beta}^{HB}/k_B$ (K)	$K_{\alpha\beta}^{HB}$ (Å^3)	*N° sites* [a)]	*Error (AAD%)*	*Source*
[P_{4444}][Ac]	318.48	9.324	3.862	406.9	3500	1900	1+1	$1.9\cdot10^{-3}$	This work

[a)] The number of sites for ILs are counted as dual sites + negative sites.

Section S6. Energy consumption for TS process

Table S3. Literature sources of the isobaric heat capacities used in this work.

Compound	Ref.	C_p estimated at 313 K [J. mol^{-1}. K^{-1}]
[$P_{666,14}$][Cl]	[7]	794.7
[$P_{666,14}$][Ac]	[8]	1113.3
[$P_{666,14}$][Br]	Estimated from [9]	1067.8
[$P_{666,14}$][MetS]	[7]	634.0
[$P_{666,14}$][Dec]	[8]	1203.9
[$P_{666,14}$][bis-2,4,4 TMPP]	[10]	1665.3

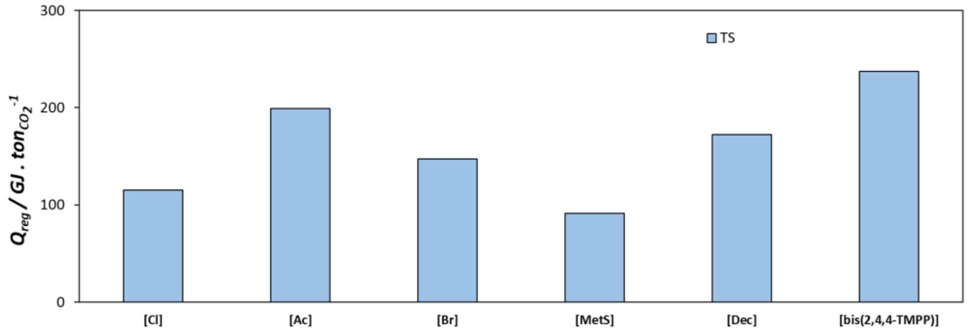

Figure S.8 – Energy consumption for a temperature swing CO_2 separation process with the conditions detailed in Table 4.

Section S7. Additional mass transfer results

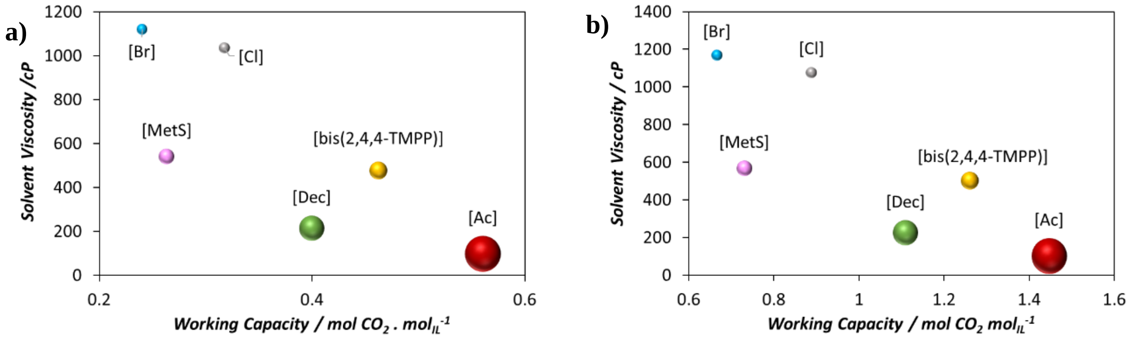

Figure S.9 – Comparative assessment of PILs performance under PS operation during the desorption process. Results were obtained using soft-SAFT simulations at a) PS operation with T = 313 K, P_{abs} = 1 MPa and P_{des} = 0.1 MPa; and b) PS operation with T = 313 K, P_{abs} = 2.5 MPa and P_{des} = 0.01 MPa. The size of the balls represents the CO_2 diffusivity in each solvent.

References

(1) Alonso, G.; Gamallo, P.; Sayós, R.; Llovell, F. Combining Soft-SAFT and COSMO-RS Modeling Tools to Assess the CO2–SO2 Separation Using Phosphonium-Based Ionic Liquids. *J. Mol. Liq.* **2020**, *297*. https://doi.org/10.1016/j.molliq.2019.111795.

(2) Supporting Information for Review Only. **2025**.

(3) Grimme, S.; Antony, J.; Ehrlich, S.; Krieg, H. A Consistent and Accurate Ab Initio Parametrization of Density Functional Dispersion Correction (DFT-D) for the 94 Elements H-Pu. *J. Chem. Phys.* **2010**, *132* (15). https://doi.org/10.1063/1.3382344.

(4) Boys, S. F.; Bernardi, F. The Calculation of Small Molecular Interactions by the Differences of Separate Total Energies. Some Procedures with Reduced Errors. *Mol. Phys.* **1970**, *19* (4), 553–566. https://doi.org/10.1080/00268977000101561.

(5) Simon, S.; Duran, M.; Dannenberg, J. J. How Does Basis Set Superposition Error Change the Potential Surfaces for Hydrogen-bonded Dimers? *J. Chem. Phys.* **1996**, *105* (24), 11024–11031. https://doi.org/10.1063/1.472902.

(6) Pena, C. A.; Soto, A.; Rodríguez, H. Tetrabutylphosphonium Acetate and Its Eutectic Mixtures with Common-Cation Halides as Solvents for Carbon Dioxide Capture. *Chem. Eng. J.* **2021**, *409* (October 2020). https://doi.org/10.1016/j.cej.2020.128191.

(7) Ferreira, A. F.; Simões, P. N.; Ferreira, A. G. M. Quaternary Phosphonium-Based Ionic Liquids: Thermal Stability and Heat Capacity of the Liquid Phase. *J. Chem. Thermodyn.* **2012**, *45* (1), 16–27. https://doi.org/10.1016/j.jct.2011.08.019.

(8) Oster, K.; Goodrich, P.; Jacquemin, J.; Hardacre, C.; Ribeiro, A. P. C.; Elsinawi, A. A New Insight into Pure and Water-Saturated Quaternary Phosphonium-Based Carboxylate Ionic Liquids: Density, Heat Capacity, Ionic Conductivity, Thermogravimetric Analysis, Thermal Conductivity and Viscosity. *J. Chem. Thermodyn.* **2018**, *121*, 97–111. https://doi.org/10.1016/j.jct.2018.02.013.

(9) Oster, K.; Jacquemin, J.; Hardacre, C.; Ribeiro, A. P. C.; Elsinawi, A. Further Development of the Predictive Models for Physical Properties of Pure Ionic Liquids: Thermal Conductivity and Heat Capacity. *J. Chem. Thermodyn.* **2018**, *118*, 1–15. https://doi.org/10.1016/j.jct.2017.10.010.

(10) Ferreira, A. G. M.; Simões, P. N.; Ferreira, A. F.; Fonseca, M. A.; Oliveira, M. S. A.; Trino, A. S. M. Transport and Thermal Properties of Quaternary Phosphonium Ionic Liquids and IoNanofluids. *J. Chem. Thermodyn.* **2013**, *64*, 80–92. https://doi.org/10.1016/j.jct.2013.04.013.
